# Removing leakage-induced correlated errors in superconducting quantum error correction

**DOI:** 10.1038/s41467-021-21982-y

**Published:** 2021-03-19

**Authors:** M. McEwen, D. Kafri, Z. Chen, J. Atalaya, K. J. Satzinger, C. Quintana, P. V. Klimov, D. Sank, C. Gidney, A. G. Fowler, F. Arute, K. Arya, B. Buckley, B. Burkett, N. Bushnell, B. Chiaro, R. Collins, S. Demura, A. Dunsworth, C. Erickson, B. Foxen, M. Giustina, T. Huang, S. Hong, E. Jeffrey, S. Kim, K. Kechedzhi, F. Kostritsa, P. Laptev, A. Megrant, X. Mi, J. Mutus, O. Naaman, M. Neeley, C. Neill, M. Niu, A. Paler, N. Redd, P. Roushan, T. C. White, J. Yao, P. Yeh, A. Zalcman, Yu Chen, V. N. Smelyanskiy, John M. Martinis, H. Neven, J. Kelly, A. N. Korotkov, A. G. Petukhov, R. Barends

**Affiliations:** 1 Department of Physics, University of California, Santa Barbara, CA USA; 2 Google, Santa Barbara, CA USA; 3 Google, Venice, CA USA; 4 Johannes Kepler University, Linz, Austria; 5 University of Texas at Dallas, Richardson, TX USA; 6 Department of Electrical and Computer Engineering, University of California, Riverside, CA USA

**Keywords:** Quantum information, Qubits

## Abstract

Quantum computing can become scalable through error correction, but logical error rates only decrease with system size when physical errors are sufficiently uncorrelated. During computation, unused high energy levels of the qubits can become excited, creating leakage states that are long-lived and mobile. Particularly for superconducting transmon qubits, this leakage opens a path to errors that are correlated in space and time. Here, we report a reset protocol that returns a qubit to the ground state from all relevant higher level states. We test its performance with the bit-flip stabilizer code, a simplified version of the surface code for quantum error correction. We investigate the accumulation and dynamics of leakage during error correction. Using this protocol, we find lower rates of logical errors and an improved scaling and stability of error suppression with increasing qubit number. This demonstration provides a key step on the path towards scalable quantum computing.

## Abstract (web summary)

Correlated errors coming from leakage out of the computational subspace are an obstacle to fault-tolerant superconducting circuits. Here, the authors use a multi-level reset protocol to improve the performances of a bit-flip error correcting code by reducing the magnitude of correlations.

## Introduction

Quantum error correction stabilizes logical states by operating on arrays of physical qubits in superpositions of their computational basis states^1–3^. Superconducting transmon qubits are an appealing platform for the implementation of quantum error correction^4–13^. However, the fundamental operations, such as single-qubit gates^14,15^, entangling gates^16–20^, and measurement^21^ are known to populate non-computational levels, creating a demand for a reset protocol^22–27^ that can remove leakage population from these higher states without adversely impacting performance in a large-scale system. Directly quantifying leakage during normal operation presents another challenge, as optimizing measurement for detecting multiple levels is hard to combine with high speed and fidelity. This calls for analysis methods that use the errors detected during the stabilizer code’s operation to find and visualize undesired correlated errors.

Here we introduce a multi-level reset gate using an adiabatic swap operation between the qubit and the readout resonator combined with a fast return. It requires only 250 ns to produce the ground state with fidelity over 99%, with gate error accurately predicted by an intuitive semi-classical model. This fidelity is achieved simultaneously on all of the first three excited states for a single parameter choice. The gate is straightforward to calibrate and robust to drift due to the adiabaticity. Further, it uses only existing hardware as needed for normal operation and readout, and does not involve strong microwave drives that might induce crosstalk, making it attractive for large-scale systems.

We benchmark the reset gate using the bit-flip error correction code^5^ and measure growth and removal of leakage in-situ. By purposefully injecting leakage, we also quantify the gate’s impact on errors detected in the code. Finally, we introduce a technique for computing the probabilities of error pairs, which allows identifying the distinctive patterns of correlations introduced by leakage. We find applying reset reduces the magnitude of correlations. We use these pair probabilities to inform the identification and correction of errors, improving the code’s performance and stability over time.

## Results

### Reset gate implementation

**Fig. 1:**
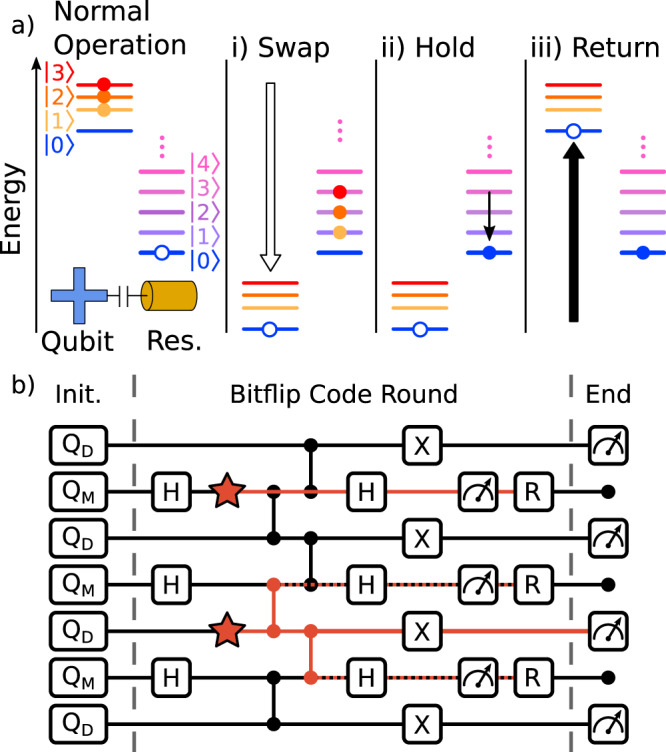
Removing leakage with reset. **a** Schematic of the multi-level reset protocol. The qubit starts with a population in its first three excited states (closed circles), with the readout resonator in the ground state (open circle). (i) The qubit is swept adiabatically past the resonator to swap excitations. (ii) Resonator occupation decays to the environment while the qubit holds. (iii) After the resonator is sufficiently depleted, the qubit returns diabatically to its operating frequency. The total duration of the reset protocol is about 250 ns. **b** Circuit for the bit-flip stabilizer code including reset (R). Measure qubits (*Q*_*M*_) cyclically apply parity measurements to neighboring data qubits (*Q*_*D*_) using Hadamard (H) and CZ gates. We add X gates to data qubits to depolarize energy relaxation error. When introducing reset, leakage errors (stars) may be removed from both measure and data qubits, either directly or via transport through the CZ gates (red lines).

The multi-level reset gate consists of the three distinct stages dubbed “swap”, “hold”, and “return” (Fig. 1a). First, we swap all qubit excitations to the resonator by adiabatically sweeping the qubit frequency to ~1 GHz below the resonator frequency. We then hold the qubit below the resonator while excitations decay to the environment. Finally, we return the qubit diabatically to its initial frequency.

Pulse engineering of the “swap” stage is critical to achieving efficient population transfer. We adopt a fast quasi-adiabatic approach^28^, where the qubit frequency changes rapidly when far detuned from the resonator level crossing but changes slowly when near the level crossing. Since the frequency changes more slowly near the level crossing than a linear ramp, the probability of a diabatic error ${{P}_{D}}^{(s)}$ can be upper bounded by a Landau-Zener transition. This gives ${{P}_{D}}^{(s)}\ll \exp \left(-{(2\pi g)}^{2}{t}_{{\rm{swap}}}/{{\Delta }}f\right) \sim 1{0}^{-3}$, where *t*_swap_ = 30 ns, Δ*f* = 2.5 GHz is the total qubit frequency change and *g* ≈ 120 MHz is the qubit-resonator coupling^29^.

The “hold” stage of the protocol is primarily described by resonator photon decay. This decay follows $\exp (-\kappa {t}_{{\rm{hold}}}) \sim 1{0}^{-3}$, with *t*_hold_ ~ 300 ns and *κ* ~ 1/(45 ns) the resonator decay rate. The qubit’s excitation number remains mostly unchanged during the hold below the resonator as Purcell decay^30^ through the resonator is small. For swap durations below 30 ns the adiabaticity of the swap transition breaks down, and the system enters the “hold” stage in a superposition of the two adiabatic eigenstates. As a result, the probability undergoes coherent Rabi oscillations, which causes an incomplete reset and manifests itself as fringes.

If a single photon remains in the qubit-resonator system, the “return” stage of the protocol can be well described by a Landau-Zener transition. Achieving diabaticity is limited by the finite bandwidth of the control system. We can estimate an effective detuning velocity ${\nu }_{r}=\frac{1}{h}\frac{d}{dt}({E}_{01}-{E}_{10})={{\Delta }}f/{t}_{r}$ using the typical ramp timescale *t*_r_ = 2 ns. The probability of the desired diabatic transition is then ${P}_{D}^{(r)}=\exp [-{(2\pi g)}^{2}/{\nu }_{r}]\approx 0.6$. This description can be further extended to the multi-photon case using the Landau-Zener chain model^31^.

Combining the semi-classical descriptions of each stage, we can identify two error channels in the reset of a single excitation. The first channel corresponds to the photon adiabatically swapping into the resonator, but then surviving over the hold time and adiabatically transitioning back to the qubit during the return. This is the dominant error channel, with probability $(1-{{P}_{D}}^{(s)}){e}^{-\kappa {t}_{\text{hold}}}(1-{P}_{D}^{(r)}) \sim 5\cdot 1{0}^{-4}$. The second channel corresponds to a failed initial swap of the qubit photon, followed by a diabatic transition during the return. The probability of this error is small, approximately ${{P}_{D}}^{(s)}{P}_{D}^{(r)}\ll 1{0}^{-4}$. The reset dynamics of the $\left|2\right\rangle$ and $\left|3\right\rangle$ states is similar, with multiple adiabatic transitions moving 2 and 3 photons to the resonator respectively, after which they undergo rapid decay.

We experimentally test our reset gate on a Sycamore processor^29^, consisting of an array of flux-tunable superconducting transmon qubits^4,32^ with tunable couplers^17,29,33,34^. Each qubit is coupled to a readout resonator with strength *g* ≈ 120 MHz, and having a frequency ~1.5 GHz below the qubit. Resonators are coupled to the outside environment through a Purcell filter^35^.

**Fig. 2:**
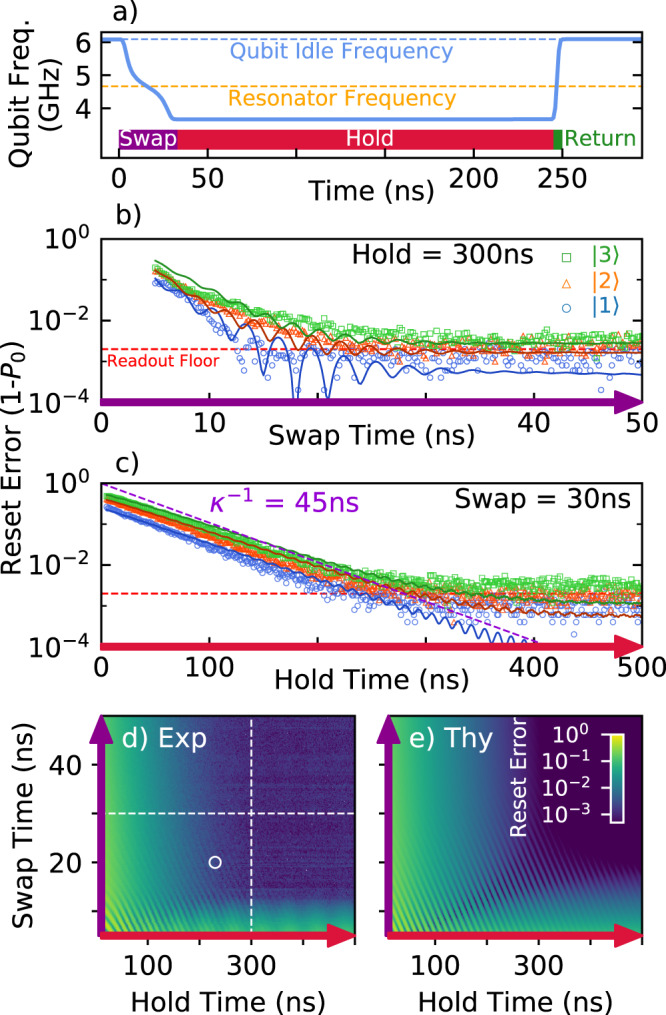
Reset gate benchmarking. **a** The qubit frequency trajectory for implementing reset consists of three stages. We plot the ground state infidelity when resetting the first three excited states of the qubit versus swap (**b**) and vs hold times (**c**). We include experimental data (points) and theory prediction (solid lines). Reset error versus swap and hold for the experiment (**d**) and theory (**e**) show a wide range of optimal parameters. Dashed white lines indicate linecuts for (**b**) and (**c**). White circle indicates the point of operation.

The reset gate is implemented using flux-tuning pulses to steer the qubit’s frequency to interact with the resonator, see Fig. 2a. The selected qubit has an idle frequency of 6.09 GHz and a nonlinearity of −200 MHz. The qubit starts at its idle frequency, moves past the resonator at 4.67 GHz, and is held 1 GHz below it, followed by a fast return to the idle frequency. We define the reset error as the likelihood of producing any state other than the ground state. The dependence of reset error on swap duration is shown in Fig. 2b for the cases when the qubit is initialized to $\left|1\right\rangle$, $\left|2\right\rangle$, and $\left|3\right\rangle$. We find that the reset error for all of the initialized states decreases until it reaches a readout visibility floor at about 30 ns swap duration. This floor of ~0.2% was also measured independently as the ground state measurement error after heralding; postselecting on a prior measurement of $\left|0\right\rangle$. This indicates that the floor is intrinsic to the measurement, not to the reset gate itself. We notice oscillations in the data which arise from an incomplete swap and are reproduced by the theoretical model results. In Fig. 2c, we keep the swap duration fixed at 30 ns and vary the hold duration. We find that the reset error decreases exponentially until it reaches the readout visibility floor, with a decay that is compatible with 1/*κ* = 45 ns. We show the landscape of the reset error for the qubit initialized in $\left|1\right\rangle$, experimentally in Fig. 2d, and the model results in Fig. 2e. For a wide choice of parameters above a minimum swap and hold duration, the ground state can be achieved with high fidelity: Experimentally we are limited by readout and theoretically the deviation from the ground state is below 10^−3^. We also note that the majority of error is favorably in the computational basis, which stabilizer codes can naturally identify and correct. The landscape involving other parameters can be found in Supplementary Note 1.

The data and model results in Fig. 2 show that one can reset a qubit within 250 ns to the ground state with an error of around 10^−3^. Moreover, the insensitivity to parameter choice, stemming from the adiabaticity of the gate, highlights the protocol’s robustness to drift and noise. This makes it amenable to use in large-scale systems. Finally, the demonstrated ability to simultaneously remove occupation from the $\left|1\right\rangle$, $\left|2\right\rangle$, and $\left|3\right\rangle$ states for a single choice of parameters makes this protocol a prime candidate for mitigating leakage in quantum error correction.

### Bit-flip code

We now benchmark this protocol in the bit-flip stabilizer code^5^, a precursor to the surface code. Here, a fast cycle of Hadamard, entangling, and measurement gates is repeated (Fig. 1b) to extract parity measurements to stabilize the logical state. We note the addition of X gates on the data qubits to depolarize energy relaxation error. Since the reset protocol is designed to unconditionally prepare the ground state, and thus remove all quantum data, we apply it only on the measure qubits immediately after readout. In the absence of reset, we apply no feedback to the measure qubit state but account for this during syndrome decoding as in ref. ^5^.

**Fig. 3:**
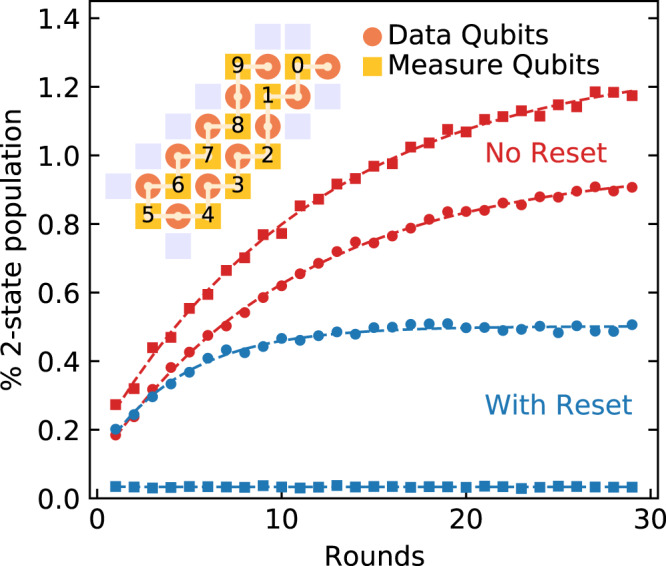
Leakage during the bit-flip code. The growth in $\left|2\right\rangle$ population vs. stabilizer code length. The circuit is run for a number of rounds and terminated with a readout sensitive to $\left|2\right\rangle$ population. The experimental data are averaged over measure or data qubits and fitted to an exponential (dashed lines) to extract rates. Further data are included in Supplementary Note 3. The inset shows the 21 qubit chain as implemented on the Sycamore device.

We implement a 21 qubit chain on a Sycamore processor (inset of Fig. 3). The qubits chosen had an average *T*_1_ near 14 μs, with their experimental parameters chosen by optimization^36^. We start by directly measuring the growth of leakage to $\left|2\right\rangle$ by running the code for a number of rounds and terminating with a measurement that can resolve $\left|2\right\rangle$ on all qubits. Each round is 955 ns long when we include reset. We note that the leakage population is subject to a different readout floor than seen in Fig. 2, as further detailed in Supplementary Note 2. We average over 40 random initial states for the data qubits, and find that the population of $\left|2\right\rangle$ grows and saturates. In the absence of reset, the measure qubits build up a larger $\left|2\right\rangle$ state population than the data qubits.

We fit a simple rate equation model and calculate the leakage (*γ*_*↑*_) and decay (*γ*_*↓*_) rates for the $\left|2\right\rangle$ state population^15^. Applying reset to the measure qubits breaks the established pattern of growth and requires a different fitting procedure, detailed in Supplementary Note 3. We find a fortyfold increase in *γ*_*↓*_ on average for measure qubits with the addition of reset. We also find a 2.4× increase in *γ*_*↓*_ on average for data qubits, indicating transport of leakage population from data to measure qubits. We understand this effect as arising naturally in our CZ gate^33^, which requires a condition that also places $\left|21\right\rangle$ and $\left|03\right\rangle$ on resonance, where the $\left|2\right\rangle$ is on the lower frequency qubit. Where a data qubit is below the measure qubit in frequency, transport of $\left|2\right\rangle$ from the data qubit to $\left|3\right\rangle$ in the measure qubit can occur, where it is subsequently removed by reset.

### Injection of leakage

**Fig. 4:**
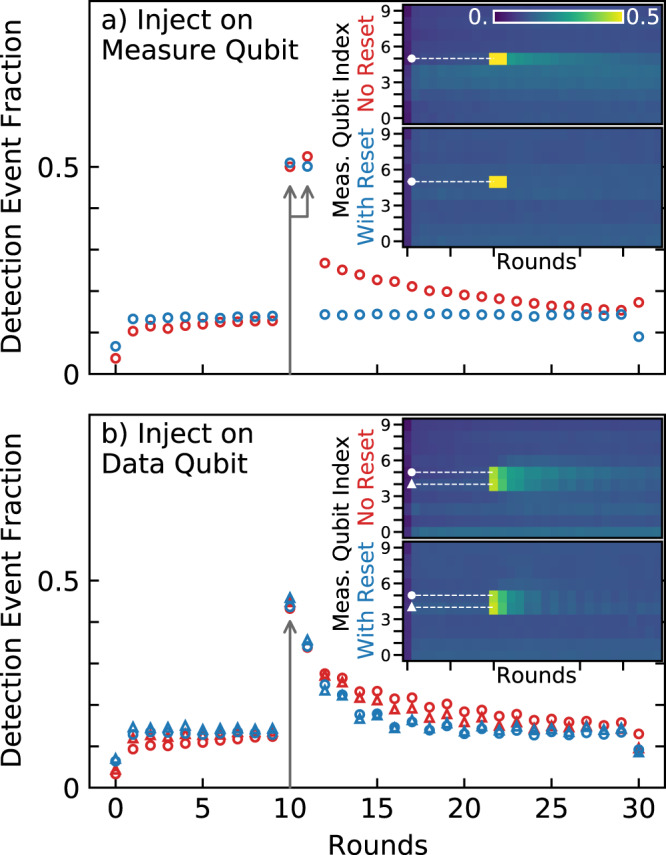
Injection of leakage. Detection event fraction when a full $\left|1\right\rangle \to \left|2\right\rangle$ rotation is inserted in round 10 after the first Hadamards **a** on measure qubit 5 and **b** on the data qubit between measure qubits 4 (circles) and 5 (triangles). Insets show the event fraction across all measure qubits, indicating the traces plotted in the main figure (dashed lines). See Fig. 3 inset for qubit locations.

To visualize the pattern of errors that leakage produces, we now inject $\left|2\right\rangle$ into the stabilizer code at specific locations. We insert a complete rotation between $\left|1\right\rangle$ and $\left|2\right\rangle$ on a single qubit immediately after the first Hadamard gates in round 10 of a 30 round experiment. As the data qubits are in either $\left|0\right\rangle$ or $\left|1\right\rangle$, and measure qubits are in an equal superposition of $\left|0\right\rangle$ and $\left|1\right\rangle$ after the Hadamard, the amount of injected $\left|2\right\rangle$ is the same for both measure and data qubits on average. Figure 4 shows the fraction of error detection events, which represents the portion of runs where a given stabilizer measurement reports an unexpected result, indicating an error occurred^5^. Injected leakage produces two distinct effects; a pair of detection events at injection, and a tail of correlated detection events over the lifetime of the leakage state. As with discrete bit-flip errors, the initial pairs of detection events appear sequentially in time for injection on measure qubits, while for data qubits both adjacent measure qubits report error (gray arrows).

The detection event fractions for all qubits are shown in the insets and cross-sections are shown in the main figure. We note that the value of the detection event fraction deviates for the first round due to initialization, and for the last round as data qubit measurements are involved^5^. As can be seen in Fig. 4a, the insertion of leakage in measure qubit 5 (see inset of Fig. 3 for its location) creates two adjacent peaks at a detection event fraction of 0.5, as the injection produces a random readout result in round 10. This is followed by a clear tail of anomalously high levels of detection events that slowly decays over many rounds, indicating errors that are correlated in time. When applying reset, the errors on all measure qubits are more uniform, and the increase in detection events for the first nine rounds becomes flattened. Importantly, the slow decay in errors is no longer visible as the detection event fraction drops to the baseline immediately after the initial pair of detection events. We also insert leakage in the data qubit between measure qubits 4 and 5, see Fig. 4b. We again notice an increase of detection events that slowly decays, now on both neighboring measure qubits. The error decreases more rapidly with reset, corroborating our prior observation that higher-level states can migrate to measure qubits. In addition, we notice a small increase in detection events around the leakage injection in qubits 3 and 6 in the case of no reset, further indicating that higher-level states can move between qubits. We notice for both cases a small odd-even oscillation in the data, which we understand as arising from the fact that the injected $\left|1\right\rangle$ to $\left|2\right\rangle$ rotation does not affect the data qubit when it is in state $\left|0\right\rangle$. Since the X gates on data qubits swap $\left|0\right\rangle$ and $\left|1\right\rangle$ in each round, we see a higher likelihood of bit error from energy relaxation in odd rounds after the injection.

The data in Fig. 4 show that the reset protocol can remove large populations of leakage in measure qubits and helps to decrease leakage in data qubits, thereby strongly suppressing time-correlated tails of detection events. This result also raises the question of how higher-level state occupations that naturally arise during the stabilizer codes lead to correlated errors.

### Leakage-induced correlations

**Fig. 5:**
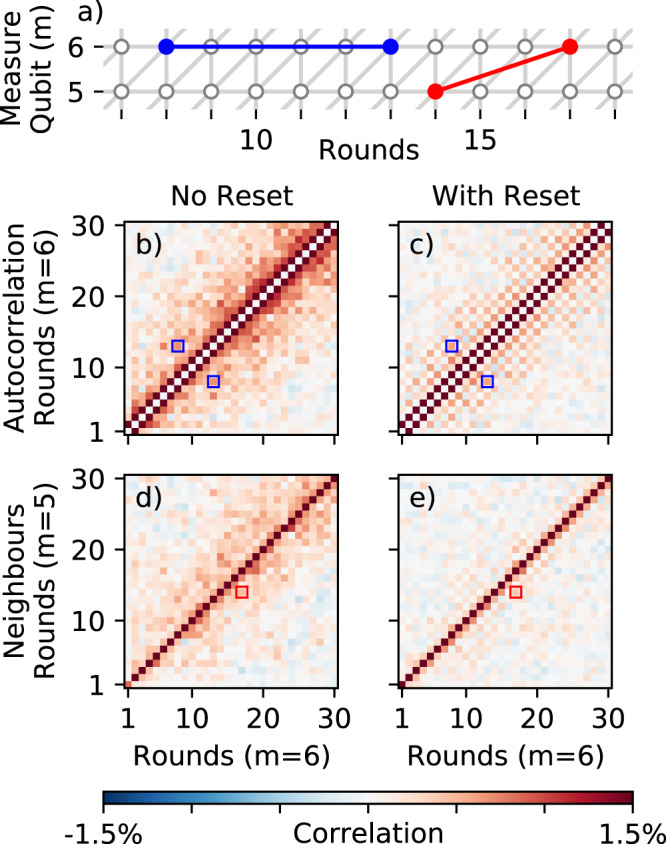
Correlations caused by leakage. *p*_*ij*_ matrices show the strength of non-local correlations in the detected errors. These undesired correlations are significantly reduced with the addition of reset. **a** The error graph for the bit-flip code, highlighting examples of non-local correlations on both space and time, indicating their corresponding *p*_*ij*_ elements below (boxes). **b**, **c** Time-correlations on measure qubit 6, with and without reset. **d**, **e** Cross-correlations between measure qubits 5 and 6, with and without reset.

To further quantify this, we analyze the correlations between detection events that arise during normal code operation using the error graph^5^, see Fig. 5a. We model detection events as arising from independent random processes that flip pairs of measurements^37^. The probability *p*_*ij*_ of the process that flips measurements *i* and *j* can be obtained from the observed correlations between detection events,

1 $${p}_{ij}=\frac{1}{2}-\frac{1}{2}\sqrt{1-\frac{4(\langle {x}_{i}{x}_{j}\rangle -\langle {x}_{i}\rangle \langle {x}_{j}\rangle )}{1-2\langle {x}_{i}\rangle -2\langle {x}_{j}\rangle +4\langle {x}_{i}{x}_{j}\rangle }},$$

where *x*_*i*_ = 1 if there is a detection event at a given measurement *i* and *x*_*i*_ = 0 otherwise. Here 〈*x*〉 denotes averaging *x* over many experimental realizations.

In Fig. 5, we visualize *p*_*ij*_ and show autocorrelations for measure qubit 6 and cross correlations between measure qubits 5 and 6. The standard error correction model assumes that detection events occur only in local pairs. For detection events occurring on the same measure qubit, we expect only correlations between adjacent rounds, corresponding to elements adjacent to the main diagonal (*p*_*i,i*±1_) of Fig. 5b, c. For detection events occurring on neighboring measure qubits, we expect only correlations between the qubits in the same round, or adjacent rounds due to the staggered placement of CZ gates. This corresponds to non-zero elements only on and immediately below the main diagonal of Fig. 5d, e. In contrast, without reset, we find that significant unexpected correlations appear (left panels), covering distances of over 10 rounds. With reset, these long-range correlations are mostly removed (right panels). This reveals an underlying checkerboard pattern that arises similarly to the aforementioned odd-even oscillations (see Supplementary Note 5).

### Logical performance and Λ_bit_

Having shown the reset protocol removes leakage and suppresses long-distance correlations, we now look at logical error rates. We run the stabilizer code to a given number of rounds, and feed the detection events into a minimum weight perfect matching algorithm^38^ that identifies and keeps track of errors to return the corrected logical state.

**Fig. 6:**
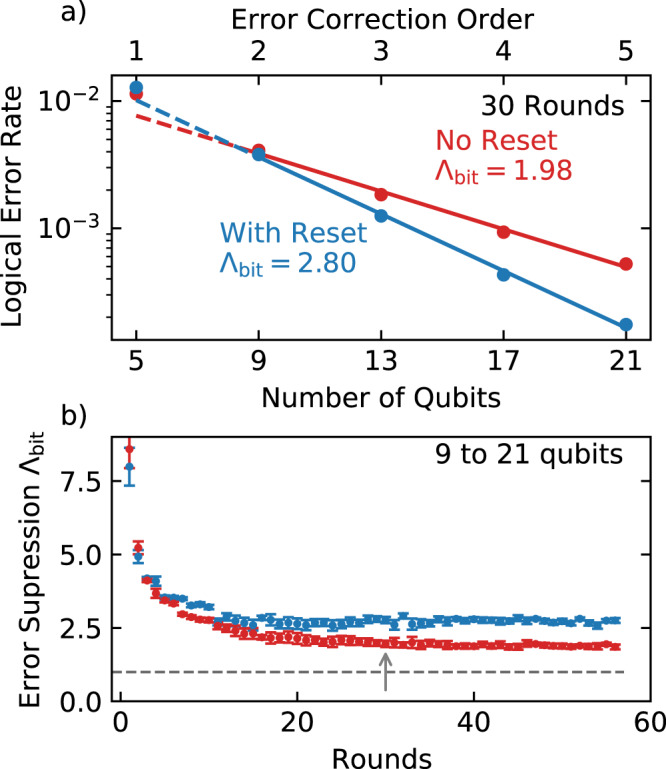
Logical code performance. **a** The logical error rate for 30 rounds vs system size. The error suppression factor Λ_bit_ is fitted to the data from nine qubits up. **b** Λ_bit_ versus code depth, showing that with reset logical error suppression is improved consistently. The error bars indicate the standard deviation error in the fit of error rate versus number of qubits. The threshold for the bit-flip code (unity) is shown as a dashed line. The arrow indicates the data in (**a**).

We perform the experiment on 21 qubits, and use subsampling to evaluate performance for smaller subsets of the code (See Supplementary Notes 6 and 7 for details). We use the *p*_*ij*_ elements to set the weights for the matching algorithm. We convert the probability of a logical error (*P*_*L*_) at a given number of rounds *k* to a logical error rate $\epsilon =[1-{(1-2{P}_{L})}^{(1/k)}]/2$^39^ for the number of rounds *k*, shown at 30 rounds in Fig. 6a. Here, the logical error rate is plotted from 5 to 21 qubits, corresponding to an error correction order of *n* = 1 to 5, meaning at least *n* + 1 errors must occur to cause a logical error. The error rate of the bit-flip code in the absence of correlations should be exponentially suppressed with $\epsilon \propto 1/{{{\Lambda }}}_{{\rm{bit}}}^{n+1}$.

We find that the logical error rate decreases with number of qubits, with an exponential dependence from 9 qubits up. The data at 5 qubits shows degraded logical performance, which we attribute to relatively narrow code width impacting the performance of syndrome decoding^40^. As including these points would reduce the quality of the fit and artificially increase the reported value of Λ_bit_, we exclude them.

We plot Λ_bit_ versus rounds in Fig. 6b. A constant logical error rate should produce a Λ_bit_ that is independent of a round number. In practice, effects including the buildup of leakage, the thermalization of data qubits, and short time boundary effects will produce a higher apparent Λ_bit_ prior to saturation. Without reset, we observe Λ_bit_ decaying over 30 rounds toward a saturation value of 1.98. With the reset, Λ_bit_ stabilizes faster, within 10 rounds, to a higher value of 2.80. Notably, error suppression is enhanced despite the time added to the cycle by reset, where data qubits are exposed to additional decoherence. This highlights the importance of removing the time-correlated errors induced by leakage, as seen in Fig. 5.

We observe the logical performance stabilizing to values of Λ_bit_ > 1, and that the addition of reset improves both the long-time performance and rate with which the code approaches this value. Moreover, we see deviations from ideal behavior where experiments are small in number of qubits or rounds. This highlights that error suppression is a property that asymptotically emerges with space and time.

In summary, we introduce a reset protocol that uses existing hardware to remove higher-level states and test it using the bit-flip stabilizer code. We show that reset mitigates leakage-induced long-time correlated errors and significantly improves logical error suppression. While optimizing gates and readout to have minimal leakage is a necessary strategy, the correlated nature of the error that leakage induces makes reset protocols critical for practical quantum error correction.

## Supplementary information

Supplementary Information

## Data Availability

The data that support the findings of this study are available from the corresponding author upon reasonable request.
