# Supplementary material for: Removing leakage-induced correlated errors in superconducting quantum error correction
Source: Nat Commun. 2021 Mar 19;12:1761. doi: 10.1038/s41467-021-21982-y (PMC7979694; doi:10.1038/s41467-021-21982-y)
Supplement: Supplementary file 1 — Supplementary Information [file 41467_2021_21982_MOESM1_ESM.pdf]

Supplementary information for "Removing leakage-induced correlated errors in superconducting quantum error correction"

M. McEwen,[1,2] D. Kafri,[3] Z. Chen,[2] J. Atalaya,[3] K. J. Satzinger,[2] C. Quintana,[2] P. V. Klimov,[2] D. Sank,[2] C. Gidney,[2] A. G. Fowler,[2] F. Arute,[2] K. Arya,[2] B. Buckley,[2] B. Burkett,[2] N. Bushnell,[2] B. Chiaro,[2] R. Collins,[2] S. Demura,[2] A. Dunsworth,[2] C. Erickson,[2] B. Foxen,[2] M. Giustina,[2] T. Huang,[2] S. Hong,[2] E. Jeffrey,[2] S. Kim,[2] K. Kechedzhi,[3] F. Kostritsa,[2] P. Laptev,[2] A. Megrant,[2] X. Mi,[2] J. Mutus,[2] O. Naaman,[2] M. Neeley,[2] C. Neill,[2] M. Niu,[3] A. Paler,[4,5] N. Redd,[2] P. Roushan,[2] T. C. White,[2] J. Yao,[2] P. Yeh,[2] A. Zalcman,[2] Yu Chen,[2] V. N. Smelyanskiy,[3] John M. Martinis,[1] H. Neven,[2] J. Kelly,[2] A. N. Korotkov,[2,6] A. G. Petukhov,[2] and R. Barends[2]

[1] *Department of Physics, University of California, Santa Barbara, CA 93106, USA*
[2] *Google, Santa Barbara, CA 93117, USA*
[3] *Google, Venice, CA 90291, USA*
[4] *Johannes Kepler University, 4040 Linz, Austria*
[5] *University of Texas at Dallas, Richardson, TX 75080, USA*
[6] *Department of Electrical and Computer Engineering, University of California, Riverside, CA 92521, USA*

(Dated: February 9, 2021)

SUPPLEMENTARY NOTE 1: RESET GATE PARAMETERS

The multi-level reset gate has five main parameters that determine its shape, shown in Fig. 2a in the main text: the swap, hold, and return durations, and two parameters that determine the swap trajectory. We calibrate the swap and hold durations as described in the main text, and we use a minimum return duration imposed by filtering of 2 ns so as to maximize $P_D^{(r)}$.

The adiabatic swap we use follows the quasi-adiabatic approach of Ref. [1]. In this approach the pulse shape $f_q(t)$ is designed in terms of the control angle θ on the Bloch sphere of states $|01\rangle$ and $|10\rangle$, $\tan(\theta) = 2g/(f_q - f_r)$, $0 < \theta < \pi$, where $f_q(t)$ is the qubit frequency, f_r is the resonator frequency, and g is the qubit-resonator coupling. However, the reset gate has to operate not only for the initial state $|1\rangle$ of the qubit, but also for the states $|2\rangle$ and $|3\rangle$, which have stronger couplings. Moreover, there are three relevant resonance conditions for these cases: $f_r = f_q$, $f_r = f_q + \eta$, and $f_r = f_q + 2\eta$, where $\eta \simeq -200\,\text{MHz}$ is the qubit nonlinearity. We therefore use a phenomenological approach and design the pulse $f_q(t)$ as in Ref. [1], but for optimized coupling and resonator frequency. We replace g and f_r with free parameters μ and f_{swap} respectively, and optimize experimental performance of the reset gate over these parameters.

For clarity, we now describe the process outlined in Ref. [1] in more detail. The pulse shape $f_q(t)$ is parametrized as $d\tilde{\theta}/dx = (\tilde{\theta}_{\text{fin}} - \tilde{\theta}_{\text{in}}) \sum_{n=1}^{3} \lambda_n [1 - \cos(2\pi n x)]$, where $\sum_{n=1}^{3} \lambda_n = 1$, $\tan(\tilde{\theta}) = 2\mu/(f_q - f_{\text{swap}})$, and $0 < \tilde{\theta} < \pi$. Here, the initial and final values of $\tilde{\theta}$ are defined as $\tan(\tilde{\theta}_{\text{in}}) = 2\mu/(f_{\text{idle}} - f_{\text{swap}})$ and $\tan(\tilde{\theta}_{\text{fin}}) = 2\mu/(f_{\text{hold}} - f_{\text{swap}})$. We also define a dimensionless natural time x, $0 \le x \le 1$, for which the Rabi frequency is constant. This dimensionless time is related to the physical time t as $t = t_{\text{swap}} \int_0^x \sin\tilde{\theta}(x')\,dx' / [\int_0^1 \sin\tilde{\theta}(x')\,dx']$. We first calculate $\tilde{\theta}(x)$ analytically, then calculate $t(x)$ numerically, and then use numerical interpolation to find $\tilde{\theta}(x(t))$. Finally, the qubit trajectory is obtained as $f_q(t) = f_{\text{swap}} + 2\mu \cot[\tilde{\theta}(t)]$. We use $\lambda_1 = 1.15$, $\lambda_2 = -0.2$, and $\lambda_3 = 0.05$, similar to the values used in Ref. [2]. We set f_{hold} to be 1 GHz below the readout resonator frequency f_r to minimize hybridization.

Supplementary Fig. 1 shows the error of the reset gate as a function of the parameter f_{swap} and the swap duration t_{swap} for the qubit initial states (a) $|1\rangle$, (b) $|2\rangle$, and (c) $|3\rangle$. For the initial state $|1\rangle$ and small values of swap duration, we see that performance is optimized near $f_{\text{swap}} = f_r$, whereas at higher values of t_{swap} the dependence is obscured by the readout floor. As expected, for the initial states $|2\rangle$ and $|3\rangle$, the optimal value of f_{swap} is higher than f_r. Nevertheless, we set $f_{\text{swap}} = f_r$; this gives an acceptable performance for all initial states at sufficiently long t_{swap}.

The parameter μ affects the slope of the pulse shape $f_q(t)$ at f_{swap}, and therefore the adiabaticity. A larger value of μ (compared with g) increases the slope near f_{swap} but decreases the slope at the sides of the pulse, thus broadening the frequency range around f_{swap} over which the slope is approximately constant. This results in a larger diabatic error for the initial state $|1\rangle$ (for which $\mu = g$ would be optimum), but decreases the error for the initial states $|2\rangle$ and $|3\rangle$, for which the first resonance occurs at $f_q + \eta = f_r$ and $f_q + 2\eta = f_r$. Supplementary Fig. 2 shows the error landscape as a function of μ and t_{swap} for the three initial states. As expected, for the initial states $|2\rangle$ and $|3\rangle$, having values of μ larger than $g = 120\,\text{MHz}$ is preferred. As a compromise, we choose $\mu = 150\,\text{MHz}$. This value not only relaxes the frequency selectivity for the crossing point, but also decreases sensitivity to noise or drift in the frequency bias.

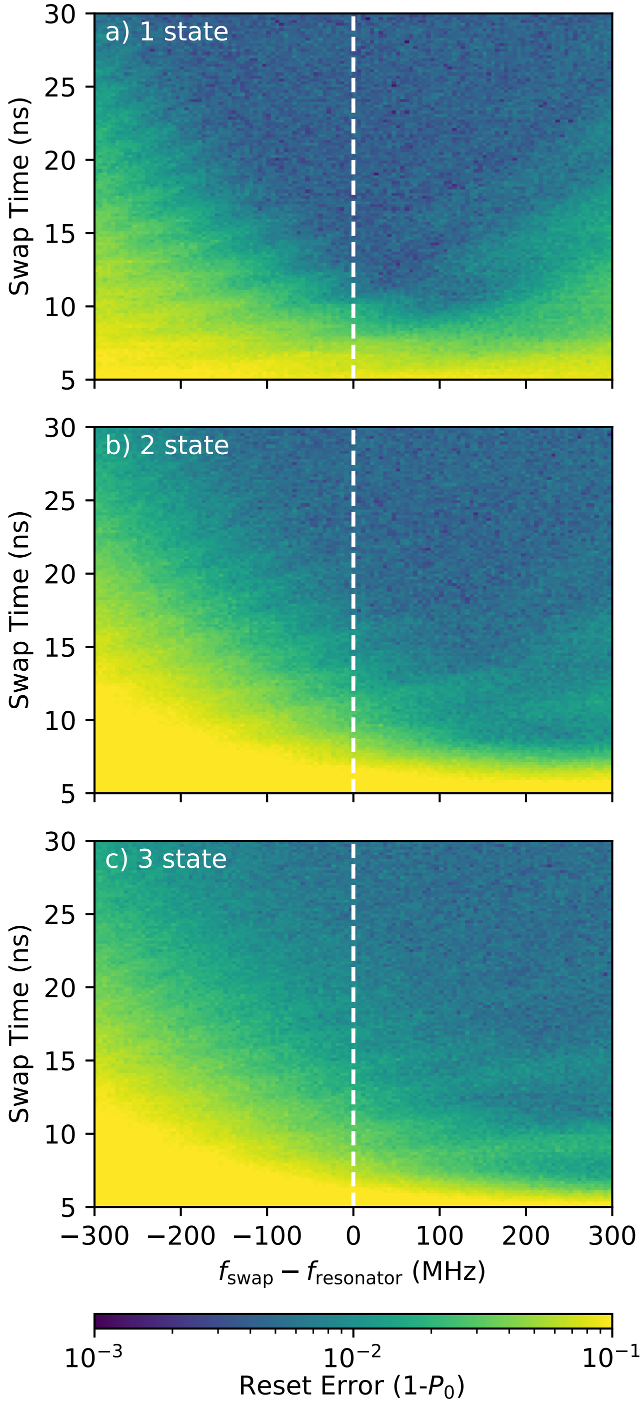

Supplementary Figure 1. **Dependence on swap frequency.** Reset performance when applied to a qubit initialized in $|1\rangle$ (a), $|2\rangle$ (b) and $|3\rangle$ (c) versus $f_{\rm swap} - f_{\rm resonator}$. For short swap lengths and on input $|1\rangle$, performance is approximately optimal for $f_{\rm swap} = f_{\rm resonator}$ (dashed line). Reset of higher states also involve transitions when the qubit is above the readout resonator due to the negative nonlinearity, producing distinct landscapes. At long swap lengths, this dependence is obscured by the readout floor, but degraded performance on $|2\rangle$ and $|3\rangle$ states is visible for $f_{\rm swap} < f_{\rm resonator}$.

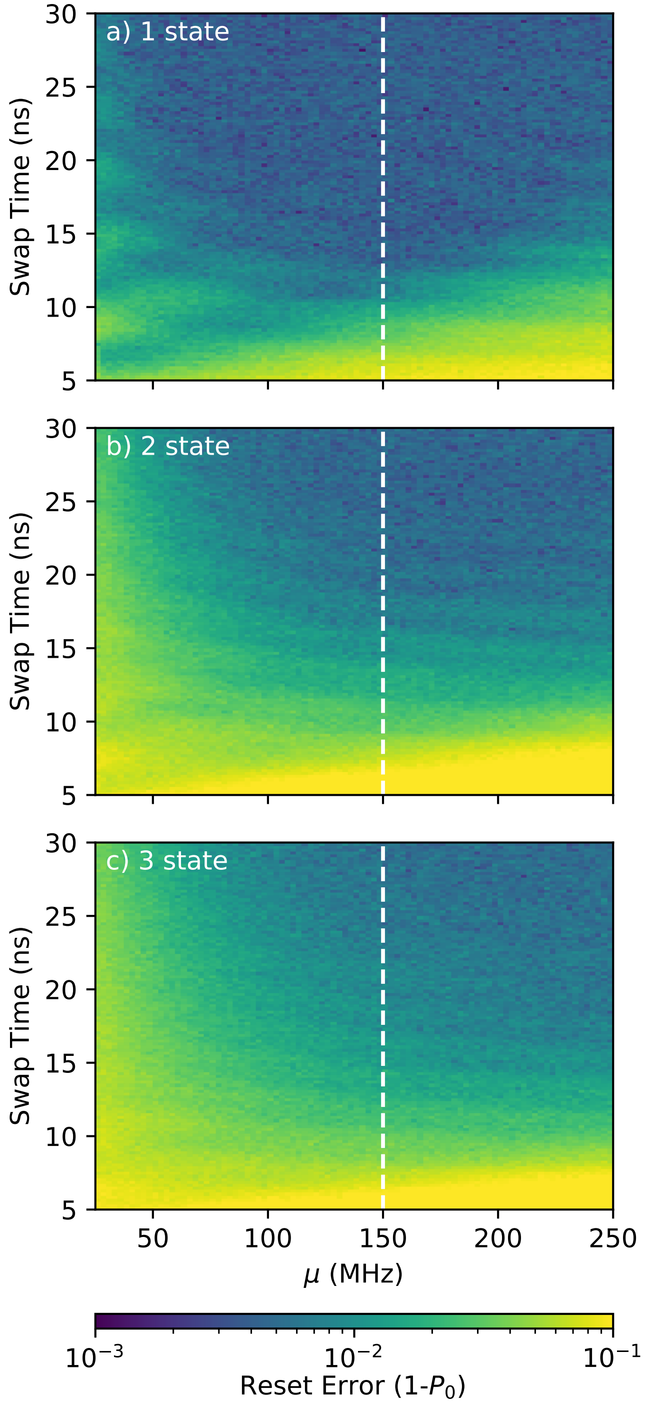

Supplementary Figure 2. **Dependence on adiabatic slope parameter.** Reset performance when applied to qubits in states $|1\rangle$ (a), $|2\rangle$ (b) and $|3\rangle$ (c) versus the slope parameter μ. For short swap lengths, performance by different input states show different profiles depending on the number of transitions involved. We choose a value of μ=150 MHz as a compromise between these three cases (dashed lines). At long swap lengths, this dependence is mostly obscured by the readout floor, but degraded performance on $|2\rangle$ and $|3\rangle$ states is visible at smaller values of μ.

SUPPLEMENTARY NOTE 2: LEAKAGE ERROR AND SUPPRESSION

We can distinguish between two kinds of error produced by the reset gate. We define the 'computational error' as the probability that the qubit is in the $|1\rangle$ state after reset, and 'leakage error' as the probability that it remains in a higher state ($|2\rangle$ and $|3\rangle$). In the context of error correction, computational error is preferred as the code naturally identifies and corrects for errors within the computational basis.

In Supplementary Fig. 3, we show reset performance separated into computational and leakage error. For short swap and hold lengths, we can see that the ratio of leakage to computational error depends on the initial state, with higher states producing more leakage error as expected. We also see a higher rate of reduction for leakage error than for computational error with hold time, reflecting the higher rate of energy relaxation from higher states. At swap and hold lengths long enough to reach the readout floor. We see that the leakage error is around 10x lower than the computational error for all states, indicating that the dominant error type is computational error.

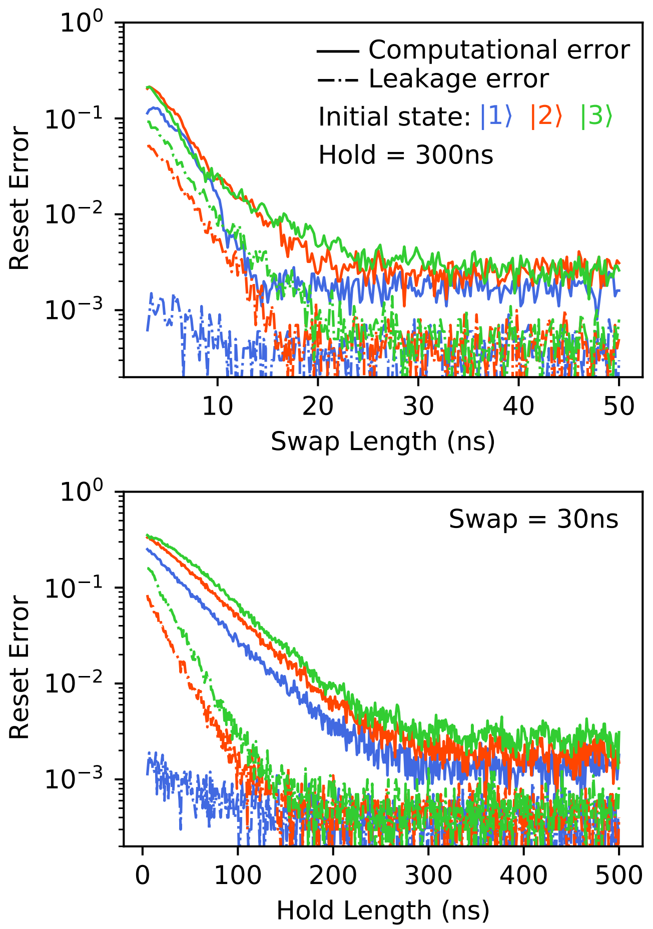

Supplementary Figure 3. **Computational vs. Leakage error.** Reset error separated into computational error (P_1, solid lines) and leakage error ($P_2 + P_3$, dash-dotted lines) for the reset gate applied to the first three excited states. We see that computational error accounts for the majority of error in all cases.

As in Fig. 3 in the main text, we can distinguish these two error types using a readout optimized for detecting higher states. A representative example of such a readout result is shown in Supplementary Fig. 4. The qubit is repeatedly prepared in $|0\rangle$, $|1\rangle$, $|2\rangle$ or $|3\rangle$, and the complex readout signal is measured and demodulated. Each shot is plotted as a single point, colored by the prepared state, allowing us to evaluate the readout fidelity for various states and to calibrate the discrimination of states. This readout was optimized to distinguish the two computational states from leakage states with high fidelity, but does not attempt to distinguish the leakage states $|2\rangle$ and $|3\rangle$ from each other.

For such a readout, we find the readout floor in Fig. 2 in the main text by heralding; performing two sequential measurements on the qubit, postselecting on $|0\rangle$ on the first measurement and calculating the fidelity of measuring $|0\rangle$ on the second measurement. We find that the

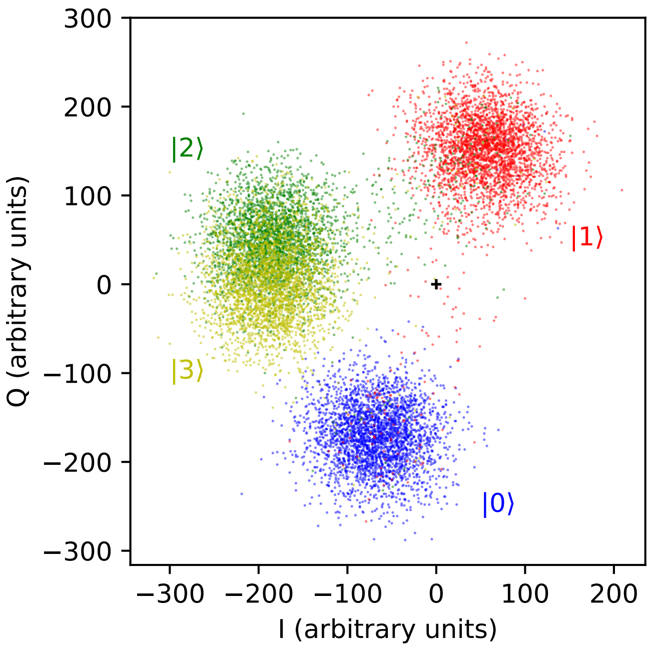

Supplementary Figure 4. **Readout for distinguishing leakage.** Demodulated in-phase (I) and quadrature (Q) components measured using a readout optimized for distinguishing computational states and leakage states. The origin is marked by a black plus. The qubit is repeatedly prepared in the $|0\rangle$, $|1\rangle$, $|2\rangle$ or $|3\rangle$ states and the raw readout signal is recorded. This illustrates our ability to distinguish the two computational states from each other and from higher level states with high fidelity.

total infidelity is around $\sim 0.2\%$ as shown in Fig. 2. We can further break this down into $\sim 0.18\%$ computational and $\sim 0.02\%$ leakage infidelity respectively, which are compatible with the values found at saturation in Supplementary Fig. 3. This also illuminates our ability to measure values for 2-state population significantly below the readout visibility floor; in Fig. 3 and Supplementary Fig. 3, we show measured values of leakage population reaching down to the floor for leakage error at $\sim 0.02\%$, below the $\sim 0.2\%$ visibility floor shown in Fig. 3.

SUPPLEMENTARY NOTE 3: LEAKAGE ACCUMULATION DURING THE BIT-FLIP CODE

In Fig. 3 of the main text, we measure the growth of leakage population during the bit-flip code using a readout similar to that shown in Supplementary Fig. 4. In Supplementary Fig. 5, we show the leakage population for each qubit over the length of the code, as well as the average that was included in Fig. 3. We note that there is significant inter-qubit variation, which we attribute to the optimization procedures we employ [3]. When applying reset, we see that all measure qubits display leakage populations around the readout floor for leakage error indicated in Supplementary Fig. 3 for all code lengths. We note that the reset protocol is capable of strongly suppressing even anomalously high rates of leakage on the measure qubits. We find the same qualitative behaviour over all qubits: The leakage populations exponentially approach saturation values of similar order over the course of the code, hence we focus our analysis on the average.

We fit the average leakage population to an exponential to extract parameters for a rate equation [4].

$$P_{|2\rangle}(k) = p_\infty \left(1 - e^{-\Gamma k}\right) + p_0 e^{-\Gamma k} \quad (1)$$

$$\Gamma = \gamma_\uparrow + \gamma_\downarrow \qquad p_\infty = \frac{\gamma_\uparrow}{\Gamma} \quad (2)$$

The rates are displayed in Supplementary Table I, showing an increase in effective leakage decay rate when reset is applied. As seen in Fig. 3 and in Supplementary Fig. 5, applying reset to the measure qubits breaks the established behaviour for growth of leakage. In order to estimate $\gamma_\downarrow$, we therefore assume a value of $\gamma_\uparrow$ equal to the case of no reset, and a value of p_∞ given by the average error for measure qubits across all rounds.

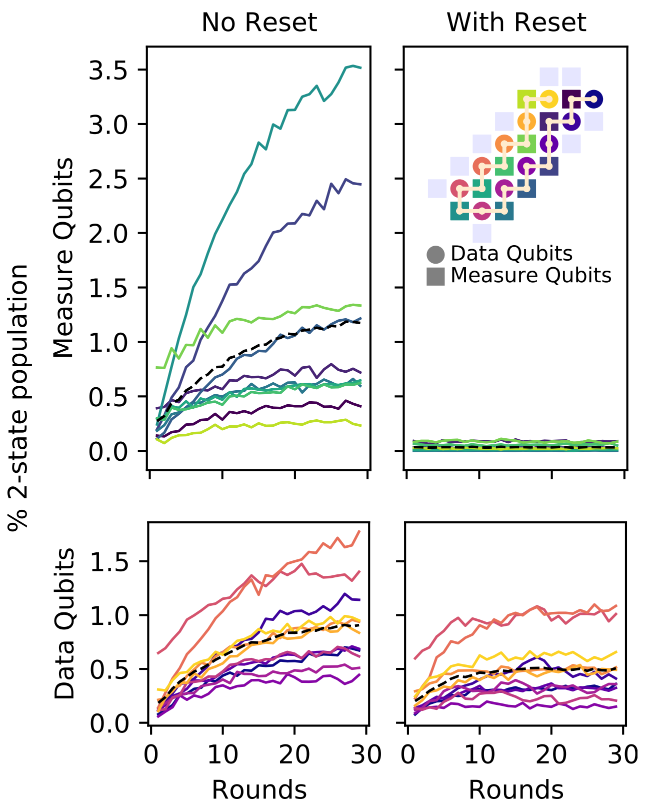

Supplementary Figure 5. **Leakage Populations during bit-flip code.** The growth in $|2\rangle$ population vs. stabilizer code length for each qubit. As in Fig. 3, the circuit is run for a number of rounds and terminated with a readout sensitive to $|2\rangle$ population. The average is included as a dashed black line. We note significant inter-qubit variation produced by calibrations, but the same qualitative behaviour across all qubits. The inset indicates the location of each qubit on the Sycamore device.

Supplementary Table I. Effective leakage growth and decay rate per stabilizer round, using Eq. 1. For the case of measure qubits with reset, $\gamma_\downarrow$ was estimated from the value of p_∞ when assuming $\gamma_\uparrow$ equal to the case of no reset (asterisk).

		$\gamma_\uparrow$	$\gamma_\downarrow$	p_∞
No Reset	Data	0.09%	9.1%	0.97%
	Measure	0.11%	8.1%	1.30%
With Reset	Data	0.11%	22.1%	0.50%
	Measure	0.11%*	328%*	0.03%*

SUPPLEMENTARY NOTE 4: THE p_{ij}-MATRIX

We first describe the model of detection events that is used to obtain Eq. 1 of the main text. We consider an error graph where each node is associated with a measure qubit and a round of the bit-flip code [5], and the edges are between *all* pairs of nodes. The state of a node

corresponds to whether an error is detected and takes values $x_i = 0$ for no detected error or $x_i = 1$ if an error was detected. The edges represent possible errors, whose occurrence flips the states of its two nodes, $x_i \to 1 - x_i$ and $x_j \to 1 - x_j$. We assume that, in each realization of the bit-flip code, an error on each edge ij occurs independently and according to a fixed probability p_{ij}, where i and j indicate the nodes connected by the error edge. The detection event at a node i will be registered ($x_i = 1$) in a realization if an odd number of errors on edges connected to the node i have occurred in that realization.

Due to the statistical independence of the errors, the statistics of nodes i and j detecting errors can be understood considering three independent processes: (1) the occurrence of an error on edge ij that flips both x_i and x_j; (2) the occurrence of an odd number of errors on edges ik with $k \neq j$, flipping x_i but not x_j; (3) the occurrence of an odd number of errors on edges kj with $k \neq i$, flipping x_j but not x_i. We can then express the averages $\langle x_i \rangle$, $\langle x_j \rangle$, and $\langle x_i x_j \rangle$ (where $\langle \cdot \rangle$ indicates averaging over realizations) in terms of the probabilities of these three processes: p_{ij}, p_i and p_j, respectively. After solving this nonlinear algebraic system of three equations, we obtain the error edge probability p_{ij} that is given in Eq. 1 of the main text. Further details are given in Ref. [6].

SUPPLEMENTARY NOTE 5: THE CHECKERBOARD PATTERN IN THE p_{ij}-MATRIX

There is a clear checkerboard pattern visible in Fig. 5c in the main text, in which the values of the correlation matrix p_{ij} for measure qubit 6 are larger for correlations spanning an odd number of rounds. For edges spanning an even number of rounds, the correlations are smaller and can even be negative. A similar but less pronounced checkerboard pattern can be seen in Fig. 5e for the cross-correlation between measure qubits 5 and 6. In fact, (b) and (c) display similar patterns, but these are visually masked by the presence of significant leakage-induced correlations. Both edges shown in Fig. 5a span odd numbers of rounds and show values larger than neighbouring values in the p_{ij} matrix.

This checkerboard pattern is caused by correlations between energy relaxations on the same data qubit. The mechanism of the correlation is illustrated in Supplementary Fig. 6. An energy relaxation event on a data qubit, $|1\rangle \to |0\rangle$, produces a pair of detection events on the neighboring measure qubits (red circles in Supplementary Fig. 6). Subsequent X-gates applied to the data qubit each round (see Fig. 1b in the main article) alternate the qubit state: $|0\rangle \to |1\rangle \to |0\rangle \to |1\rangle \to \ldots$. As a result, the qubit can relax again 1, 3, 5, ... rounds later, while relaxation after 2, 4, 6, ... rounds is unlikely. This creates a positive correlation between the errors separated

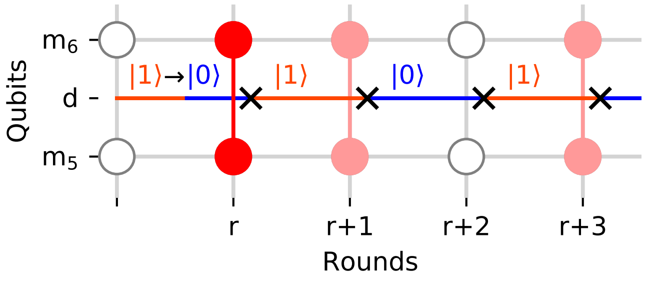

Supplementary Figure 6. **Odd-even periodicity of energy relaxation events.** The state of a data qubit d is flipped each round by an X gate (black). An energy relaxation event $|1\rangle \to |0\rangle$ in d in round r produces detection events (red circles) in the neighboring measure qubits m_5 and m_6. As the next energy relaxation event can occur only when d is in $|1\rangle$, future energy relaxation errors will be preferentially separated by an odd number of rounds from the initial event (pink circles), producing an alternating pattern of correlations.

by an odd number of rounds and negative correlation for separation by an even number of rounds, producing the checkerboard pattern. The correlations gradually decay with increasing separation. The checkerboard pattern is more pronounced in Fig. 5c as a single measure qubit is affected by both neighbouring data qubits, while in Fig. 5e the checkerboard pattern is caused by only the one data qubit between the measure qubits 5 and 6.

SUPPLEMENTARY NOTE 6: STATISTICS AND POSTSELECTION IN THE BIT-FLIP CODE

When benchmarking performance in the bit-flip code, we average over a large number of realizations, including over randomly chosen initial states for the data qubits. For the leakage populations shown in Fig. 3 of the main text, we chose 20 random initial bitstrings for the data qubits, and repeated the experiment 5000 times for each bitstring. For data shown in Figs. 4, 5 and 6, we chose 40 random bitstrings and repeated the experiment 1000 times for each bitstring for 40 000 total realizations. However, the probabilities of logical errors are smaller at low numbers of rounds, requiring additional averaging to reduce statistical error. For runs with 10 or fewer rounds, we therefore chose 100 random initial bitstrings and took 10 000 repetitions at each bitstring, for 1 000 000 total realizations.

Over these large numbers of runs, we see a small number of short events where the detection fractions are significantly elevated compared to the average [6]. We are investigating these effects. These events are not representative of the normal functioning of the device and so

we postselect them out using the following procedure. We calculate the logical error for each time-ordered realization and then calculate a moving average of the logical error over 30 realisations. The this average is typically below 3%, but during events the moving average can reach 50%. We choose a threshold of 25% to identify the start and end of an event. We remove 500 realizations before the start and 500 realizations after the end of each event, typically removing around 1200 realizations in total. This procedure removes approximately 0.8% of the data.

SUPPLEMENTARY NOTE 7: SUBSAMPLING FOR ANALYSIS OF SCALING PERFORMANCE

A central assumption in quantum error correction is that the logical performance of a code should scale exponentially with number of qubits. To analyse this scaling, instead of running multiple experiments at different numbers of qubits, we use subsampling to extract performance at lower orders from from experiments consisting of larger number of qubits, as in Ref. [5].

The 21-qubit code is 5th order fault tolerant, meaning it can correct up to 5 simultaneous X errors. This code has three possible 17-qubit subsets which are each 4th order fault tolerant. For each of these 17-qubit subsets, we can discard the appropriate data from a run of the 21-qubit code and infer the performance at 4th-order fault tolerance. Averaging over all subsets at each lower order gives more accurate estimation of the scaling performance than running separate experiments at each lower order. It naturally provides a large number of instances at low order, avoids introducing variation due to calibration drift over time and is significantly less experimentally taxing. This technique is discussed in greater detail in Section IV of the Supplementary Materials in Ref. [5].

[1] J. M. Martinis and M. R. Geller, Fast adiabatic qubit gates using only σ_z control, Phys. Rev. A **90**, 022307 (2014).

[2] R. Barends, J. Kelly, A. Megrant, A. Veitia, D. Sank, E. Jeffrey, T. C. White, J. Mutus, A. G. Fowler, B. Campbell, *et al.*, Superconducting quantum circuits at the surface code threshold for fault tolerance, Nature **508**, 500 (2014).

[3] P. V. Klimov, J. Kelly, J. M. Martinis, and H. Neven, The snake optimizer for learning quantum processor control parameters (2020), arXiv:2006.04594 [quant-ph].

[4] Z. Chen, J. Kelly, C. Quintana, R. Barends, B. Campbell, *et al.*, Measuring and suppressing quantum state leakage in a superconducting qubit, Phys. Rev. Lett. **116**, 020501 (2016).

[5] J. Kelly, R. Barends, A. G. Fowler, A. Megrant, E. Jeffrey, T. C. White, D. Sank, J. Y. Mutus, B. Campbell, Y. Chen, Z. Chen, B. Chiaro, A. Dunsworth, I. C. Hoi, C. Neill, P. J. J. O'Malley, C. Quintana, P. Roushan, A. Vainsencher, J. Wenner, A. N. Cleland, and J. M. Martinis, State preservation by repetitive error detection in a superconducting quantum circuit, Nature **519**, 66 (2015).

[6] Z. Chen *et al.*, Exponential suppression of bit or phase flip errors with repetitive error correction (2021), arXiv:2102.06132 [quant-ph].
